# Association between height loss and cardiovascular disease in the Korean elderly

**DOI:** 10.1038/s41598-022-06594-w

**Published:** 2022-02-15

**Authors:** Soo Jung Choi, Rugyeom Lee, Yewon Na, In Cheol Hwang, Jaehun Jung

**Affiliations:** 1 Department of Family Medicine, Gil Medical Center, Gachon University College of Medicine, ADD 21, Namdong-daero 774 beon-gil, Namdong-gu, Incheon, 21565 South Korea; 2 Artificial Intelligence and Big-Data Convergence Center, Gil Medical Center, Gachon University College of Medicine, Incheon, South Korea; 3 Graduate School of Public Health, Seoul National University, Seoul, South Korea; 4 Department of Preventive Medicine, Gachon University College of Medicine, 38-13, Dokjeom-ro 3, Incheon, 21565 South Korea

**Keywords:** Cardiovascular diseases, Epidemiology, Outcomes research

## Abstract

Shorter people are at risk for cardiovascular disease (CVD), but data remain limited. This study sought to determine whether height loss is associated with an increased incidence of CVD. From the Korean National Health Insurance Service—Senior database (2002–2015), data of 134,952 individuals with available information on height loss was obtained. Height loss as percentages was measured 3–5 years from the baseline height. To assess hazard ratios for CVD incidence, multivariable Cox proportional hazard regression models were used before and after applying propensity score matching. The unmatched cohort consisted of 109,546 participants without height loss (< 1%): 20,208 participants with 1–2% height loss, and 5126 participants with ≥ 2% height loss. During a median follow-up period of 6.5 years (interquartile range, 3.7–8.5 years), 21,921 were newly diagnosed with CVD. Adults with height loss of > 2% had a greater risk of incident CVD than those with no height loss. This finding was statistically significant both in the original- and propensity score-matched cohorts. The increased risk for ischemic stroke was significant in the male subgroups, in line with degree of height loss. Overall, height loss is associated with an increased risk of subsequent ischemic stroke in Korean men.

## Introduction

There is solid evidence regarding the increased risk of cardiovascular disease (CVD) in shorter people^1^; however, further studies are warranted to elucidate the underlying mechanism. Several prospective investigations with a sufficient number of cases and/or long follow-up periods have been conducted, but they do not draw a clear conclusion regarding the relationship between height loss and CVD incidence. The relationship is further complicated by the shared pathophysiology between CVD and osteoporosis^2^.

To explore this issue, longitudinal studies with sequential height measurements are warranted. One study^3^ followed 4213 men in the UK aged between 60 and 79 years. The researchers measured participants’ height changes after a period of 20 years. After a mean follow-up period of 6 years, compared to the group with a height loss of < 1 cm, the group with a height loss of ≥ 3 cm had a higher risk of CVD mortality (*P* = 0.02) and incidence of CVD (*P* = 0.03). Another study from the UK^4^ analyzed the data of 3802 men and 1615 women (aged 45 to 69 years) who had their height remeasured after an interval of 12 years. During the mean follow-up of 7.4 years, 69 cases of coronary heart diseases had occurred in the male participants and were associated with greater height loss (95% confidence interval [CI], 1.00–1.53). Recently, a Japanese study with 2498 atomic bomb survivors reported that marked height loss in middle age was an independent predictor for CVD mortality^5^.

The aforementioned studies had the following limitations. First, the data was only just within the limits of statistical significance, meaning no clear relationship between amount of height loss and CVD was described. Second, the researchers did not consider any osteoporotic events that may have critically impacted height loss. Third, they did not guarantee the generalizability in terms of sex, age, or health compared to the general population or other ethnic populations. Thus, this study aimed to investigate the relationship between height loss and CVD in a nationally representative large cohort from the Korean National Health Insurance Service database, with consideration of potential confounders including osteoporosis.

## Methods

### Study design and participants

**Figure 1:**
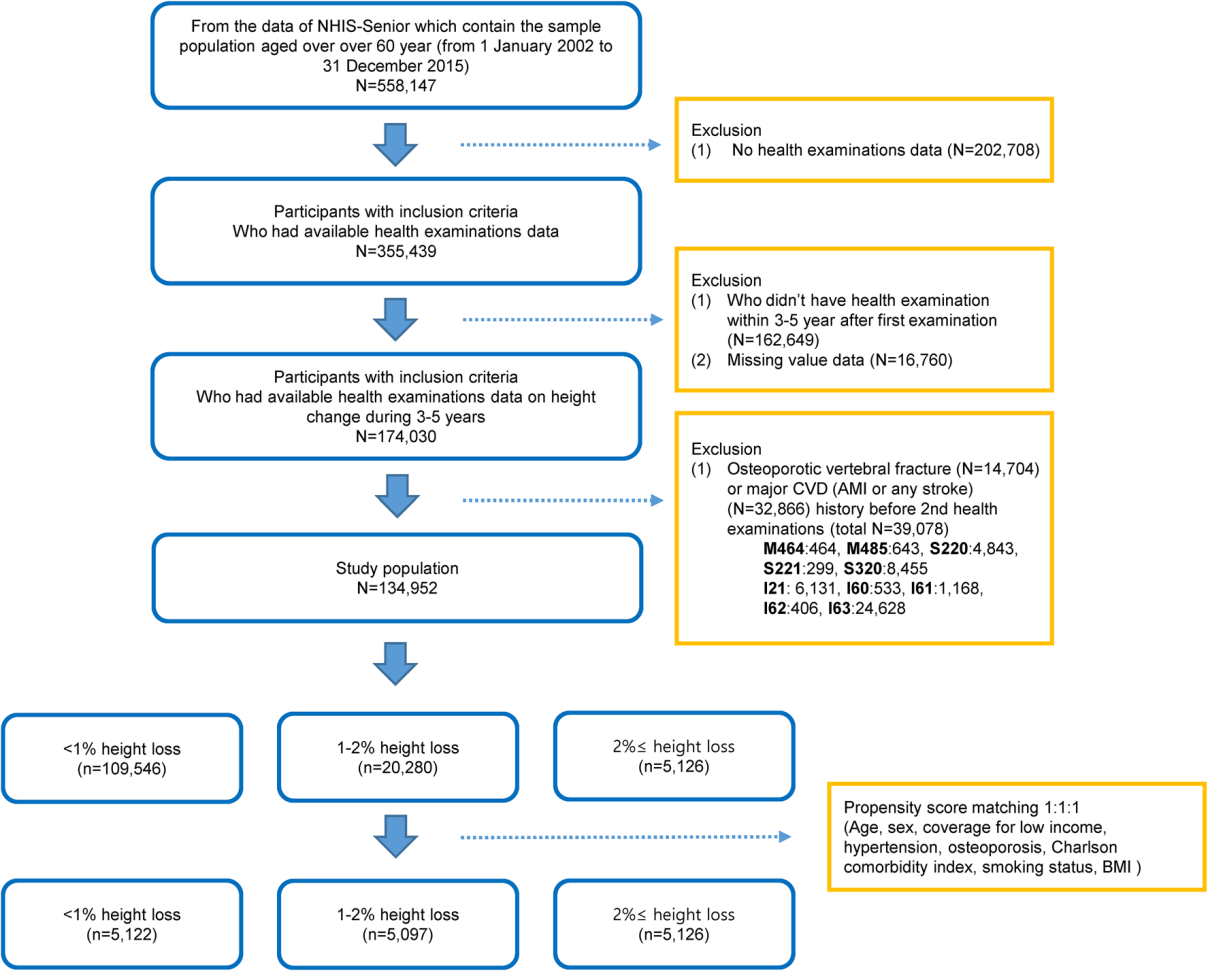
Flow diagram showing the selection process for participants. AMI, Acute Myocardial Infarction; BMI, body mass index; CVD, cardiovascular disease; NHIS, National Health Insurance Service. Created using PowerPoint 2016 (Microsoft Corporation, Redmond, WA, USA).

We used the National Health Insurance Service-Senior (NHIS-Senior) cohort^6^, provided by the Korean NHIS. The NHIS-senior cohort consists of a 10% random sample based on national claims data over 60 years old since 2002 in Korea, and has been tracked until 2015. The NHIS manages the national public health insurance program in which all health-care providers need to submit medical claims for reimbursement. NHIS subscribers receive biennial, mandatory health examinations starting from the age of 40 years. The NHIS data records demographic profile, health insurance claims, the death and disability registries, and national health check-up data. The International Classification of Diseases-10th Revision (ICD-10) was used to identify cases of morbidity and mortality. Representative 14-year (2002–2015) longitudinal data are provided to public health researchers if approved by the official review committee. We selected 174,030 individuals aged over 60 years, who had available data on height change over a period of 3–5 years. Among these, we excluded patients who had a history of osteoporotic vertebral fracture (ICD codes, M48.4, M48.5, S22.0, S22.1, S32.0; n = 14,704) or major CVD (i.e., acute myocardial infarction or any stroke; n = 32,866). Finally, 134,925 participants were enrolled as the unmatched whole cohort in this study (Fig. 1).

### Ethical approval statement

The Korean National Health Insurance Corporation provided anonymized data to the researchers after approving the study protocol (No. NHIS-2020–2-004). This study was approved by the Institutional Review Board of Gachon University Gil Medical Center (IRB No. GCIRB2019-370). Participant consent was waived by the ethics committee because the data involved routinely collected medical data that was processed anonymously at all stages. All study methods were carried out based on the Declaration of Helsinki.

### CVD outcomes and covariates

The primary outcome of this study was a newly diagnosed CVD during the follow-up period which was from the index date. The index date was recorded as the time of the second height measurement that was used to calculate height loss. Incidence of CVD included cases of acute myocardial infarction (ICD code, I21) and any stroke (ICD codes, ischemic, I63; hemorrhagic, I60–62) (Supplementary Table 1). Cases of CVD were defined as at least one outpatient visit or admission > 1, with the main or 1st subcode as one of the diagnoses.

In Korea, regular national checkups are performed every 1–2 years. At 3–5 years from the first screening, height measurements are made at the screening, and cases in which a decrease in height was confirmed were categorized and defined as within 1%, 1–2%, or > 2%. We used relative height loss, which was calculated as a percentage from the baseline height, because baseline height is associated with CVD risk^1^. Regarding information from the health checkup data, the most recent data (nearest the index date) were selected; these included height, weight, low income (medical aid benefits), and self-reported smoking status (never, ex-, or current). Body mass index (BMI, kg/m^2^) was calculated, and comorbidities such as hypertension and osteoporosis (ICD codes, M80–M82) were also assessed (Supplementary Table 1). The Charlson Comorbidity Index (CCI) was used to control for comorbid conditions^7^ (Supplementary Table 2).

### Propensity score matching (PSM)

We also used propensity score matching to reduce potential confounders and to balance the baseline covariates of the groups^8^. The propensity score is the probability that an individual will lose height, based on their individual characteristics. Individual propensities for height loss were estimated by performing a logistic regression analysis of age, sex, coverage for low income, hypertension, osteoporosis, smoking status, CCI, and BMI. A ‘‘greedy nearest-neighbor’’ algorithm was used to match patients in the groups in a 1:1:1 ratio^9^. Adequacy of matching was assessed by comparing propensity score densities, distribution, and standardized mean differences (Supplementary Table 3). This approach calculates the mean population differences of the groups and is more meaningful than assessing* P* values from t tests^10^. After propensity score matching, there were 5122 adults with no height loss (< 1%) and matched adults with some height loss (5097 with 1–2% height loss and 5126 with ≥ 2% height loss) (Fig. 1).

### Statistical analysis

Cohorts were followed up from the index date, until outcomes occurred or until the study period ended on December 31, 2015. Cox proportional hazards regression models were applied to calculate the hazard ratios (HRs) and 95% CIs for the CVD outcomes^11^. We used a global goodness-of-fit test proposed by Schoenfeld to test the proportional hazard assumption^12^. Adjusted curves for cumulative hazards were estimated using the multivariable Cox Proportional Hazard model for CVD outcomes and height loss in three categories. In the multivariable analysis, the covariates presented above were used. For stroke outcomes, the CHA_2_DS_2_–VASc score^13^ was applied in the Cox models, instead of the CCI score (Supplementary Table 4). Two-sided P values < 0.05 were considered statistically significant. Statistical tests were performed using SAS version 9.4 (SAS Institute, Cary, NC, USA).

## Results

Participant characteristics in relation to degree of height loss are described in Table 1. Individuals with greater height loss were more likely to be women, ≥ 75 years old, receive medical aid, have osteoporosis, and present a high CCI score.

**Table 1:** Baseline characteristics of study participants by degree of height loss.

	Height loss (%)			
	< 1	1–2	≥ 2	*P *value
Number	109,546	20,280	5126	
Age at entry, years	71.7 ± 4.9	72.4 ± 5.1	74.0 ± 5.8	< 0.001
< 75	84,150 (76.8)	14,618 (72.1)	3046 (59.4)	< 0.001
≥ 75	25,396 (23.2)	5,662 (27.9)	2080 (40.6)	
**Sex**				
Men	52,768 (48.2)	8780 (43.3)	1225 (23.9)	< 0.001
Women	56,778 (51.8)	11,500 (56.7)	3901 (76.1)	
Body mass index, kg/m^2^	23.8 ± 3.1	23.9 ± 3.3	23.7 ± 3.4	< 0.001
**Coverage for low income**				
No	109,047 (99.5)	20,160 (99.4)	5090 (99.3)	0.003
Yes	499 (0.5)	120 (0.6)	36 (0.7)	
**Hypertension**				
No	51,828 (47.3)	9,690 (47.8)	2359 (46.0)	0.074
Yes	57,718 (52.7)	10,590 (52.2)	2767 (54.0)	
**Osteoporosis**				
No	89,945 (82.1)	16,184 (79.8)	3758 (73.3)	< 0.001
Yes	19,601 (17.9)	4,096 (20.2)	1368 (26.7)	
**Smoking status**				
Never	84,670 (77.3)	16,114 (79.5)	4443 (86.7)	< 0.001
Ex-	11,024 (10.1)	1,738 (8.6)	244 (4.8)	
Current	13,852 (12.6)	2,428 (11.9)	439 (8.5)	
CCI score	2.86 ± 2.07	2.90 ± 2.08	2.91 ± 2.10	0.009
< 3	53,554 (48.9)	9,727 (48.0)	2439 (47.6)	0.014
≥ 3	55,992 (51.1)	10,553 (52.0)	2687 (52.4)	

*CCI* Charlson Comorbidity Index.

Data are presented as mean ± standard deviation or number (%).

**Figure 2:**
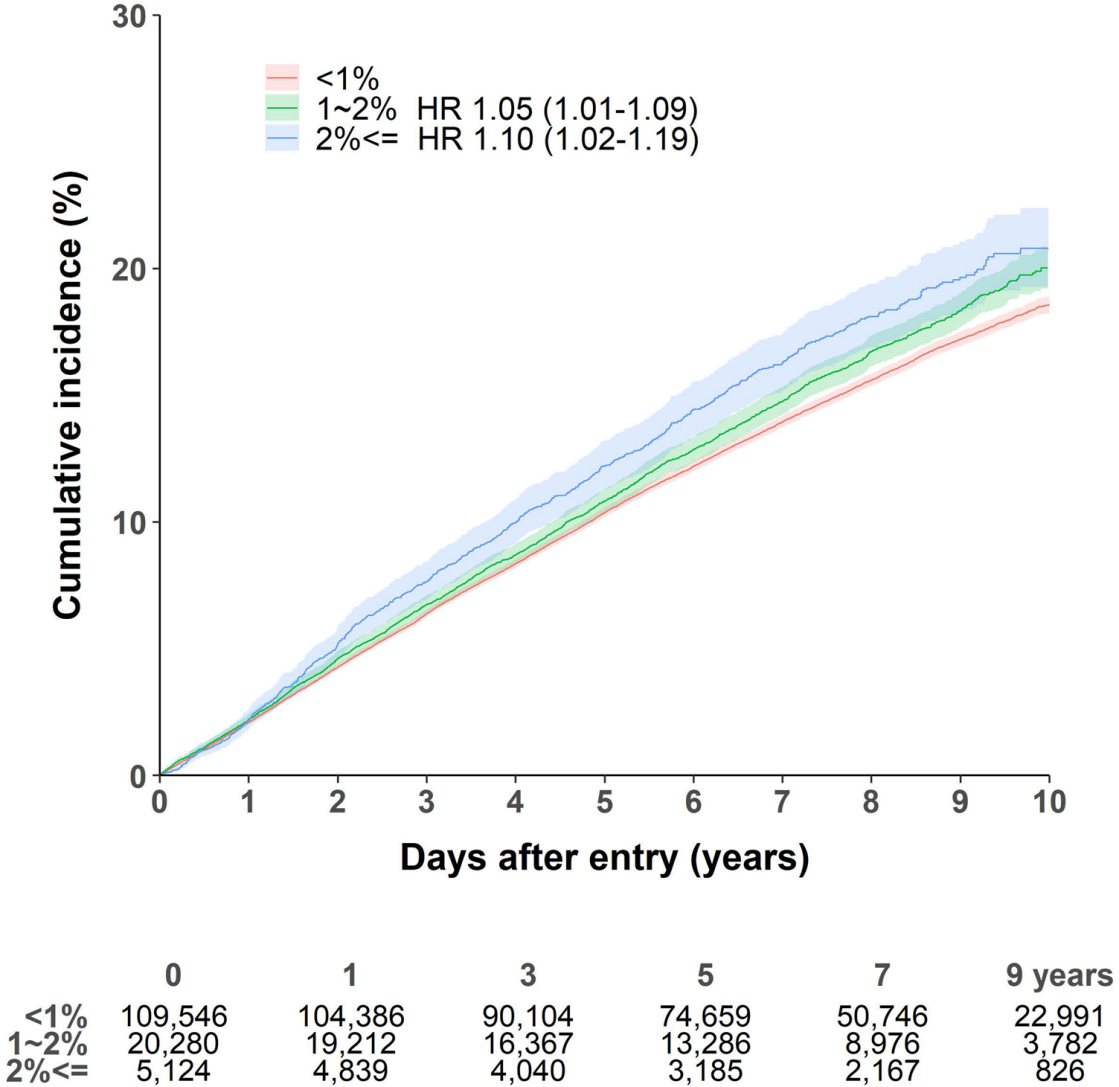
Risk of incident ischemic stroke by degree of height loss. Adjusted for age (continuous), sex, hypertension, osteoporosis, coverage for low income, smoking status, CHA_2_DS_2_–VASc score (continuous), and body mass index (continuous). Created using SAS software (version 9.4; SAS Institute Inc., Cray, NC, USA).

The result of the minimally adjusted model is presented in Supplementary Table 5. Table 2 shows the association between height loss and incident CVD. During a median follow-up period of 6.5 years (interquartile range, 3.7–8.5 years), 21,921 participants were newly diagnosed with CVD (2,577 acute myocardial infarctions, 1916 hemorrhagic strokes, and 17,575 ischemic strokes). Compared to individuals with no height loss (< 1% height loss), those with height loss ≥ 2% had an increased risk of CVD in both the original (HR, 1.11; 95% CI, 1.04–1.19) and PSM cohorts (HR, 1.93; 95% CI, 1.73–2.16). However, this association was only statistically significant in regard to ischemic stroke (Table 3 and Fig. 2). Further, risk for incident ischemic stroke was significantly correlated with degree of height loss among men (Table 3).

**Table 2:** Factors associated with incidence of cardiovascular diseases^a^, including the degree of height loss.

	Original cohort					PSM cohort				
	Cases	Participants	HR^b^	95% CI	*P *values	Cases	Participants	HR^b^	95% CI	*P *values
**Height loss (%)**										
< 1	17,631	109,546	Ref			493	5122	Ref		
1–2	3384	20,280	1.05	1.01–1.09	0.014	511	5097	1.06	0.93–1.20	0.376
≥ 2	906	5126	1.11	1.04–1.19	0.002	906	5126	1.93	1.73–2.16	< 0.001
Age, per 1-year			1.04	1.04–1.05	< 0.001			1.04	1.03–1.05	< 0.001
**Sex**										
Women	11,127	72,179	Ref			1303	11,674	Ref		
Men	10,794	62,773	1.19	1.15–1.23	< 0.001	607	3671	1.59	1.41–1.79	< 0.001
**Coverage for low income**										
No	21,755	134,297	Ref			1894	15,248	Ref		
Yes	166	655	1.72	1.47–2.00	< 0.001	16	97	1.41	0.86–2.30	0.176
**Hypertension**										
No	9202	63,877	Ref			762	7053	Ref		
Yes	12,719	71,075	1.29	1.26–1.33	< 0.001	1148	8292	1.32	1.19–1.45	< 0.001
**Osteoporosis**										
No	18,139	109,887	Ref			1463	11,299	Ref		
Yes	3782	25,065	1.00	0.96–1.04	0.982	447	4046	0.95	0.85–1.07	0.396
**Smoking status**										
Never	16,956	105,227	Ref			1589	13,371	Ref		
Ex-	1803	13,006	0.89	0.84–0.93	< 0.001	101	714	0.97	0.78–1.21	0.785
Current	3162	16,719	1.23	1.18–1.28	< 0.001	220	1,260	1.33	1.13–1.56	< 0.001
BMI, per 1-kg/m^2^			1.00	0.99–1.00	0.59			0.99	0.98–1.01	0.231
CCI, per 1-point			1.09	1.08–1.10	< 0.001			1.14	1.12–1.17	< 0.001

*BMI* body mass index, *CCI* Charlson Comorbidity Index, *HR* hazard ratio, *CI* confidence interval, *PSM* propensity score matching.

^a^Including acute myocardial infarction and any stroke.

^b^From the multivariate Cox regression model.

**Table 3:** Subgroup analysis for incident cardiovascular diseases by the degree of height loss (reference, group with height loss of < 1%).

	Height loss of 1–2%		Height loss of ≥ 2%	
	HR^a^ (95% CI)	*P *value	HR^a^ (95% CI)	*P *value
**Outcomes**				
Acute myocardial infarction	1.02 (0.92–1.13)	0.663	1.19 (0.99–1.35)	0.067
Hemorrhagic stroke^b^	1.04 (0.93–1.16)	0.454	1.03 (0.84–1.26)	0.795
Ischemic stroke^b^	1.05 (1.01–1.09)	0.024	1.10 (1.02–1.19)	0.010
**In ischemic stroke**^b^				
Men	1.07 (1.01–1.13)	0.033	1.26 (1.10–1.45)	0.001
Women	1.03 (0.98–1.09)	0.271	1.06 (0.97–1.16)	0.171
< 75 years	1.06 (1.01–1.11)	0.023	1.10 (1.00–1.21)	0.060
≥ 75 years	1.03 (0.95–1.10)	0.510	1.12 (1.00–1.26)	0.043

^a^From the multivariable Cox regression models with adjustment for age (continuous), sex, hypertension, osteoporosis, smoking status, coverage for low income, Charlson Comorbidity Index score (continuous), and body mass index (continuous).

^b^Adjusted for CHA_2_DS_2_–VASc score instead of Charlson Comorbidity Index score.

## Discussion

Prevention of CVD is a major public concern due to its increasing prevalence and resulting social burden^14^, and the need for better stratification of CVD risks prevails. Among the CVD risk factors, height is recently focused upon as it is known to be inversely associated with CVD outcomes^1^. Additionally, a number of studies have suggested that it is simple and economically viable for clinicians to monitor a patient’s change in height as a measure of health problems^15^. In this nationwide large-scale population-based study, we found that height loss is associated with CVD morbidity. These results suggest that height shrinkage is an informative, and easy to measure, indicator of worsening cardiovascular health at the population level, when more detailed measures might not be available.

Although age-related decline in height is a common observation, its possible association with CVD outcomes has rarely been studied. In prior studies^3–5^ on this issue, the small number of cases with height loss, outcome events, or lack of a clearly specified population could give rise to aberrant results, which must therefore be considered and interpreted cautiously. Of note, height shrinkage is greatest among taller individuals—who are known to be at low risk of CVD—as well as older age groups and women^16^. Thus, a lack of consideration of baseline height leads to an attenuation bias in the height-CVD association. We believe that despite the great height loss and/or long-term follow-up duration, a lack of significant results in previous studies was due to the dilution effect between greater height loss and lower baseline CVD risk in tall people. We analyzed a large-scale, nationwide cohort, which included both men and women, as well as young and elderly people, using a proportional indicator for height loss.

The causality between height loss and CVD outcomes remains to be further elucidated^2^, but longitudinal designs as used in the present study indicate that height loss might be a direct cause rather than a result of CVD. Given that osteoporotic consequences are highly linked to height loss^17^, we excluded a priori patients with vertebral fracture and only considered those with osteoporosis during the follow-up. Thus, our study included a limited number of cases of height loss caused by intervertebral disc degeneration; osteoarthritic conditions of the spine, hip, or knee; or weakness of the back muscles^18^. Paravertebral muscle weakness may reflect sarcopenia^19^. The increased risk of CVD associated with height loss may reflect poor muscular strength and skeletal muscle mass loss, both of which have been shown to be predictors of mortality^20^. Moreover, it has been established that women experience a greater degree of height decline over time than men, with one of the reasons being their vulnerability to osteoporosis and/or broken bones, and while height loss in women reflects the decrease in bone density, the cause of height loss in men has not yet been reported^21^.

Significance of height loss in cases of ischemic stroke was partially explained by the outstanding incidence of ischemic stroke in our cohort, compared with that of hemorrhagic stroke or myocardial infarction. Additionally, from the traditional mechanistic perspective, higher luminal pressure and increased shearing stress from height loss, particularly in the cerebral artery, may accelerate the progression of atherosclerosis^22^, which leads to ischemic stroke. Our subgroup analysis further showed that the correlation between height loss and incident ischemic stroke remained robust among men, which could be partially explained by the relative contribution of height loss to incident stroke (Supplementary Table 6). Our results suggest that height loss in elderly men could be an indicator of impending ischemic stroke and should be an important consideration for physicians. Furthermore, future research considering body composition would be helpful to elucidate the possible mechanism of the relationship between height loss and CVD and gender difference of this association.

Several limitations in the present study should be acknowledged. First, given that recruitment was based on the database of health examinations (the overall participation rate was 65.3%), the generated data cannot be considered free from potential healthy-user bias. If people who had not received a health screen had been included in the analysis, the significance of the results would be rather more remarkable because they would be more vulnerable to height loss and CVD development. This phenomenon might also be applied for underdiagnosis of osteoporosis in older men. Second, there may have been measurement inconsistencies as height was measured at different institutions. However, this non-differential misclassification of height loss would rather dilute the real effect. Third, some unmeasured confounding factors could remain. Early-life conditions, particularly nutrition, have been established to play an important role in shaping subsequent cardiovascular health^23^. Moreover, spine misalignment that can have an impact on the association^24^ was not considered in this study. A hospital-based study or expansion of clinical trial cohort that could capture those detailed clinical data is needed to confirm our findings.

Despite the limitations, this study contributes to the literature with its large-scale cohort sample and examination of detailed nation-wide health data. To clarify the association between height loss and cardiovascular events, a genetic or twin-track approach is needed. Further interventional research is needed to disentangle the effects of height loss prevention strategies (e.g., weight bearing exercises or anti-osteoporotic agents) on CVD incidence. Serial measurements of stature are highly feasible in clinical practice and may be useful as an indicator of cardiovascular health in the primary care setting, particularly in men.

## Supplementary Information

Supplementary Information.

## Data Availability

The datasets generated during and/or analyzed during the current study are available from the corresponding author on reasonable request.
