# Supplementary material for: Association between height loss and cardiovascular disease in the Korean elderly
Source: Sci Rep. 2022 Feb 15;12:2551. doi: 10.1038/s41598-022-06594-w (PMC8847618; doi:10.1038/s41598-022-06594-w)
Supplement: Supplementary file 1 — Supplementary Information. [file 41598_2022_6594_MOESM1_ESM.pdf]

SUPPLEMENTARY MATERIAL

Association between height loss and cardiovascular disease in the Korean elderly

Soo Jung Choi, MD, PhD[1¶], Rugyeom Lee, PhD[2¶], Yewon Na, MPH[2,3¶], In Cheol Hwang, MD, PhD[1]**, Jaehun Jung, MD, PhD[2,4]*

[1]*Department of Family Medicine, Gil Medical Center, Gachon University College of Medicine, Incheon, South Korea.*

[2]*Artificial Intelligence and Big-Data Convergence Center, Gil Medical Center, Gachon University College of Medicine, Incheon, South Korea.*

[3]*Graduate School of Public Health, Seoul National University, Seoul, South Korea*

[4]*Department of Preventive Medicine, Gachon University College of Medicine, Incheon, South Korea.*

[¶] Drs. Choi, Lee and Na contributed equally to this work as a first author.

Short title: Height Loss and CVD

***Corresponding author:**
Jaehun Jung, MD, PhD
Department of Preventive Medicine, Gachon University College of Medicine
38-13, Dokjeom-ro 3, Incheon, 21565, Korea.
Tel: +82-10-6359-3201
Fax: +82-32-458-2608
E-mail: eastside1st@gmail.com
**** Co-Corresponding author:**
In Cheol Hwang, MD, PhD
Department of Family Medicine, Gil Medical Center, Gachon University College of Medicine
ADD 21, Namdong-daero 774 beon-gil, Namdong-gu, Incheon 21565, Korea.
Tel: +82-32-460-3354
Fax: +82-32-460-3354
E-mail: spfe0211@gmail.com.

Supplementary Table 1. Diagnostic codes for major comorbid diseases.

Supplementary Table 2. International Classification of Diseases 10th Revision (ICD-10) mapping for Charlson Comorbidity Index.

Supplementary Table 3. Standardized mean difference of the variable for propensity-score matching

A. Standardized mean difference of the continuous variable for propensity-score matching

B. Standardized mean difference of categorical variables for propensity-score matching

Supplementary Table 4. International Classification of Diseases, Tenth Revision (ICD-10), mapping for CHA2DS2-VASc score

Supplementary Table 5. Age- and sex-adjusted model for the effect of height loss on incidence of cardiovascular diseases

Supplementary Table 6. Factors associated with incident stroke by sex, including the degree of height loss

Supplementary Table 1. Diagnostic codes for major comorbid diseases.

Diseases	International Classification of Diseases 10th Revision codes
Hypertension	I10–I15
Osteoporosis	M80–M82
Cardiovascular deaths	I00–I99
Acute myocardial infarction	I21
Hemorrhagic stroke	I60–I62
Ischemic stroke	I63

Supplementary Table 2. International Classification of Diseases 10th Revision (ICD-10) mapping for Charlson Comorbidity Index.

**Diseases**	**ICD-10 codes**	**Weight**
Myocardial infarction	I21, I22, I252	1
Congestive heart failure	I43, I50, I099, I110, I130, I132, I255, I420, I425, I426, I427, I428, I429, P290	1
Peripheral vascular disease	I70, I71, I731, I738, I739, I771, I790, I792, K551, K558, K559, Z958, Z959	1
Cerebrovascular disease	G45, G46, I60 ~ I69, H340	1
Dementia	F00 ~ F03, G30, F051, G311	1
Chronic obstructive pulmonary disease	J40 ~ J47, J60 ~ J67, I278, I279, J684, J701, J703	1
Connective tissue disease	M05, M32 ~ M34, M06, M315, M351, M353, M360	1
Peptic ulcer disease	K25 ~ K28	1
Mild liver disease	B18, K73, K74, K700, K701 ~ K703, K709, K717, K713, K714, K715, K760, K762 ~ K764, K768, K769, Z944	1
Diabetes without complications	E100, E101, E106, E108 ~ E111, E116, E118 ~ E121, E126, E128 ~ E131, E136, E138 ~ E141, E146, E148, E149	1
Diabetes with complications	E102 ~ E105, E107, E112 ~ E115, E117, E122 ~ E125, E127, E132 ~ E135, E137, E142 ~ E145, E147	2
Paraplegia and hemiplegia	G81, G82, G041, G114, G801, G802, G830 ~ G834, G839	2
Renal disease	N18, N19, N052 ~ N057, N250, I120, I131, N032 ~ N037, Z490, Z491, Z492, Z940, Z992	2
Cancer	C00 ~ C26, C30 ~ C34, C37 ~ C41, C43, C45 ~ C58, C60 ~ C76, C81 ~ C85, C88, C90 ~ C97	2
Moderate or severe liver disease	K704, K711, K721, K729, K765, K766, K767, I850, I859, I864, I982	3
Metastatic carcinoma	C77 ~ C80	6
Acquired immune deficiency syndrome (AIDS)	B20 ~ B22, B24	6

Supplementary Table 3. Standardized mean difference of the variable for propensity-score matching

A. Standardized mean difference of the continuous variable for propensity-score matching

	**Before matching**					**After matching**				
	<1% (n=109,546)	1–2% (n=20,280)	≥2% (n=5,126)	Standardized difference	P-value	<1% (n=5,122)	1–2% (n=5,097)	≥2% (n=5,126)	Standardized difference	P-value
	Mean (SD)	Mean (SD)	Mean (SD)			Mean (SD)	Mean (SD)	Mean (SD)		
Age	71.7 (4.87)	72.4 (5.11)		0.1338	0.0001	74.02 (5.75)	73.94 (5.61)		-0.0158	0.0004
	71.7 (4.87)		74.05 (5.78)	0.4733	0.0002			74.05 (5.78)	0.0031	0.0004
BMI	23.76 (3.09)	23.89 (3.27)		0.0432	0.0001	23.71 (3.23)	23.87 (3.32)		0.0488	0.0004
	23.76 (3.09)		23.70 (3.36)	-0.0186	0.0002			23.70 (3.36)	-0.0027	0.0004
CCI	2.86 (2.07)	2.90 (2.08)		0.0207	0.0001	2.89 (2.07)	2.85 (2.01)		-0.0169	0.0004
	2.86 (2.07)		2.91 (2.1)	0.0245	0.0002			2.91 (2.1)	0.0054	0.0004

BMI, body mass index, CCI, charlson comorbidities index; SD, standard deviation.

B. Standardized mean difference of categorical variables for propensity-score matching

	Before matching					After matching				
	<1% (n=109,546)	1–2% (n=20,280)	≥2% (n=5,126)	Standardized difference	P-value	<1% (n=5,122)	1–2% (n=5,097)	≥2% (n=5,126)	Standardized difference	P-value
Sex	52768 (48.2)	8780 (43.3)		-0.1084	0.0001	1224 (23.9)	1222 (24.0)		0.0024	0.0007
	52768 (48.2)		1225 (23.9)	-0.5982	0.0003	1224 (23.9)		1225 (23.9)	0.0000	0.0007
Coverage for low income	499 (0.5)	120 (0.6)		0.1450	0.0032	26 (0.5)	35 (0.7)		0.1676	0.0205
	499 (0.5)		36 (0.7)	0.2401	0.0091	26 (0.5)		36 (0.7)	0.1801	0.0203
Ex-smoker	11,024 (10.1)	1,738 (8.6)		-0.0411	0.0001	233(4.5)	237(4.6)		-0.0113	0.0004
Current smoker	13,852 (12.6)	2,428 (11.9)				421(8.2)	400(7.8)			
Ex-smoker	11,024 (10.1)		244 (4.8)	-0.1953	0.0002	233(4.5)		244 (4.8)	0.0155	0.0004
Current smoker	13,852 (12.6)		439 (8.5)			421(8.2)		439 (8.6)		

Hypertension	57,718 (52.7)	10,590 (52.2)		-0.0104	0.0001	2768 (54.0)	2757 (54.1)		0.0011	0.0005
	57,718 (52.7)		2,767 (54.0)	0.0286	0.0003	2768 (54.0)		2767 (54.0)	-0.0014	0.0005
Osteoporosis	19,601 (17.9)	4,096 (20.2)		0.0825	0.0001	1357 (26.5)	1321 (25.9)		-0.0164	0.0006
	19,601 (17.9)		1,368 (26.7)	0.2829	0.0003	1357 (26.5)		1368 (26.7)	0.0055	0.0006

Supplementary Table 4. International Classification of Diseases, Tenth Revision (ICD-10), mapping for CHA2DS2-VASc score

**$CHADS_2$ score list**	**ICD-10 codes or condition**	**Score**
Congestive heart disease	I110, I50, I971	1
Hypertension	I10 ~ I15	1
Diabetes mellitus	E10 ~ E15	1
Stroke/TIA/thromboembolism	I63, I64, G45, I260, I269, I74	2
Vascular disease[a]	I21, I22, I252, I700, I701, I702, I708, I709, I739	1
Sex	Female	1
Age	65≤age<75	1
	75≤age	2
Maximum score		9

TIA: transient ischemic attack

[a]including prior myocardial infarction, peripheral artery disease, aortic plaque

Supplementary Table 5. Age- and sex-adjusted model for the effect of height loss on incidence of cardiovascular diseases

	Original cohort				
	Cases	Participants	Hazard ratio	95% confidence interval	*P*-values
Height loss (%)					
<1	17,631	109,546	Reference		
1–2	3,384	20,280	1.06	1.02–1.10	0.010
≥2	906	5,126	1.14	1.06–1.21	0.001
Age, per 1-year			1.05	1.04–1.06	<0.001
Sex					
Women	11,127	72,179	Reference		
Men	10,794	62,773	1.23	1.18–1.28	<0.001

Supplementary Table 6. Factors associated with incident stroke by sex, including the degree of height loss

	Men					Women				
	Cases	Participants	HR[a]	95% CI	*P*-values	Cases	Participants	HR[a]	95% CI	*P*-values
Height loss (%)										
<1	7,256	52,768	Ref.			7,338	56,778	Ref.		
1–2	1,297	8,780	1.07	1.01–1.13	0.033	1,518	11,500	1.03	0.98–1.09	0.271
≥2	208	1,225	1.26	1.10–1.45	0.001	548	3,901	1.06	0.97–1.16	0.171
Age, per 1-year			1.04	1.03–1.04	<0.001			1.02	1.01–1.02	<0.001
Coverage for low income										
No	8,722	62,539	Ref.			9,309	71,758	Ref.		
Yes	48	234	1.55	1.17–2.06	0.003	95	421	1.86	1.52–2.27	<0.001
Hypertension										
No	4,012	32,164	Ref.			3,529	31,263	Ref.		
Yes	4,758	30,159	1.13	1.08–1.19	<0.001	5,875	40,916	1.15	1.10–1.21	<0.001
Osteoporosis										
No	8,466	60,549	Ref.			6,495	49,338	Ref.		
Yes	304	2,224	0.99	0.88–1.11	0.865	2,909	22,841	1.04	1.00–1.09	0.612
Smoking status										
Never	5,137	35,193	Ref.			9,092	70,034	Ref.		
Ex-	1,394	12,525	0.87	0.82–0.92	<0.001	55	481	0.93	0.71–1.21	0.563
Current	2,239	15,055	1.17	1.11–1.23	<0.001	257	1,664	1.23	1.08–1.39	0.001
BMI, per 1-kg/m^2			1.00	0.99–1.01	0.976			0.99	0.99–1.00	0.016
CHA2DS2–VASc score, per 1-point			1.18	1.16–1.20	<0.001			1.17	1.53–1.19	<0.001

BMI, body mass index; HR, hazard ratio; CI, confidence interval.

[a]From the multivariable Cox regression models with adjustment for age (continuous), hypertension, osteoporosis, smoking status, coverage for low income, CHA2DS2–VASc score (continuous), and body mass index (continuous).
